# An unexplained fetal intracranial hemorrhage with extensive and multifocal hemorrhagic lesions: A case report

**DOI:** 10.1097/MD.0000000000029335

**Published:** 2022-06-24

**Authors:** Baorong Gao, Li Zhang, Qiang Wei

**Affiliations:** Department of Obstetrics and Gynaecology, West China Second University Hospital, Key Laboratory of Birth Defects and Related Diseases of Women and Children of the Ministry of Education, Sichuan University, No.20, Section 3, Renmin Nan Lu, Chengdu, Sichuan Province, China.

**Keywords:** case report, diagnosis, fetal intracranial hemorrhage, predisposing factors

## Abstract

**Rationale:**

Fetal intracranial hemorrhage (ICH) is an extremely rare complication of pregnancy, with subsequent neurological sequelae or fetal death. The diagnosis of fetal ICH is primarily based on ultrasound or magnetic resonance imaging.

**Patient concerns:**

An asymptomatic woman at 31 weeks of gestation was referred for a detailed anomaly scan because routine fetal ultrasonography showed suspected fetal ICH.

**Diagnoses:**

Fetal ICH with extensive and multifocal hemorrhagic lesions was diagnosed by ultrasound and magnetic resonance imaging imaging and finally confirmed by postmortem examination.

**Interventions:**

The woman opted for pregnancy termination after medical consultation. Labor was induced by mifepristone and rivanol infusion.

**Outcomes:**

The patient delivered a stillborn male infant weighing 1522 g. We tried our best to screen the possible etiology contributing to fetal ICH; unfortunately, no evidence of obvious causes or predisposing factors was identified.

**Lessons:**

Medically unexplained massive fetal ICH may cause an unfavorable prognosis, and prompt termination of pregnancy is appropriate, although there is no consensus on the optimal mode of delivery.

## 1 Introduction

Intracranial hemorrhage (ICH) refers to bleeding within the cerebral ventricles, including the brain parenchyma and surrounding meningeal spaces. Hemorrhages may occur in utero, and an estimated incidence of 1 in 10 000 pregnancies has been reported.^[1]^ Although advances in prenatal ultrasonography and magnetic resonance imaging (MRI) have increased the ability to identify fetal ICH,^[2,3]^ fetal ICH remains a rare prenatal event. The most relevant risk factors for fetal ICH include maternal trauma and fetal coagulation disorders^[1,3,4]^ however, in many cases, predisposing risk factors have not been identified. According to the four-grade classification based on the location and severity of hemorrhage,^[5,6]^ the poor outcomes have often been linked to higher-grade ICH.

## 2 Case report

A 27-year-old nulliparous woman was referred for a detailed anomaly scan because routine fetal ultrasonography revealed suspected ICH at 31 weeks of gestation. The patient reported an uneventful pregnancy. Her medical, family, and obstetric histories were noncontributory, and she denied drug ingestion.

**Figure 1:**
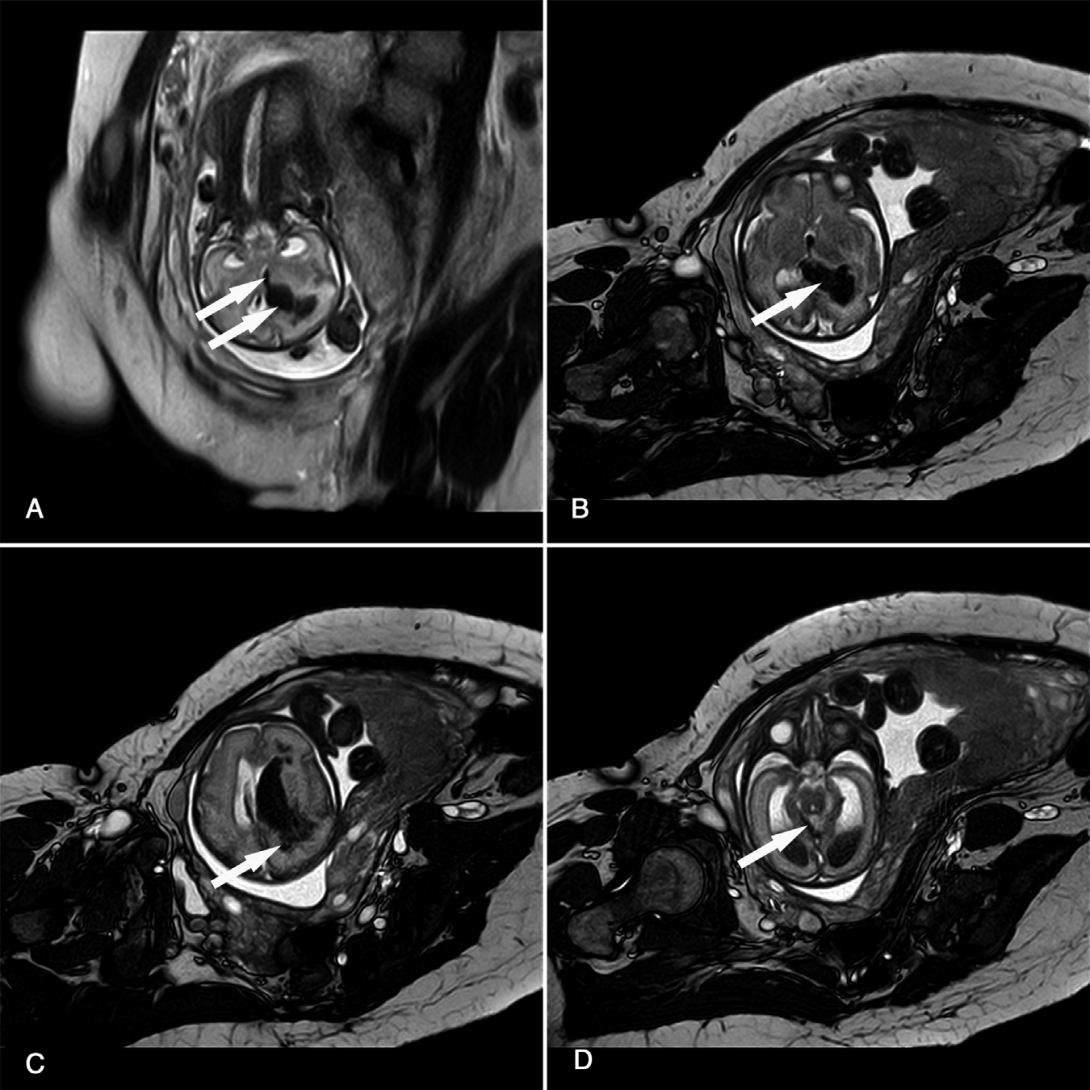
Intrauterine T2-weighted magnetic resonance images (MRI) demonstrating massive and multifocal hemorrhagic lesions (arrows) (A) the third ventricle and bilateral ventricle (B) bilateral ventriculomegaly (C) the right frontal and parietal lobes (D) tentorial subdural hemorrhage.

Obstetric ultrasonography revealed ventriculomegaly with an irregular choroid plexus. The pulsatility index (PI) of the fetal middle cerebral artery was 1.5. Fetal MRI was performed to confirm ventriculomegaly with diffuse and multifocal hypointense masses in the bilateral ventricle, third ventricle, and frontal and parietal lobes, with the midline slightly shifted to the left side. A small subdural hematoma under the tentorium of the cerebellum was observed (Fig. 1). According to the classification modified by Ghi et al,^[3]^ fetal ICH was classified as grade III or greater.

The patient underwent further investigation, including toxoplasmosis, rubella, cytomegalovirus, and herpes, parvovirus B19, assays for isoimmune and alloimmune thrombocytopenia, platelet count, prothrombin time, partial thromboplastin time, plasminogen, von Willebrand factor, factor V Leiden, anticoagulant protein S, activated protein C, and antiplatelet antibodies, all of which appeared normal. After consultation with neonatology and pediatric neurology specialists and discussion of possible neurologic sequelae, the patient decided to terminate the pregnancy. Labor was induced by mifepristone and rivanol infusion. She delivered a stillborn male infant weighing 1522 g. Postmortem examination of both the fetus and placenta was obtained after parental consent was obtained. The mother was discharged on postpartum day 2.

Autopsy confirmed the prenatal diagnosis, and all fetal analysis tests were reported with normal results.

## 3 Ethic statement

The requirement for institutional review board approval was waived owing to the retrospective nature of the study. Written informed consent was obtained from the patient for the publication of this case report.

## 4 Discussion

ICH is thought to be uncommon in prenatal fetuses, because fetal ICHs are always without any clinical symptoms, and most of them are detected fortuitously by routine obstetric ultrasonography scan; however, evaluation of fetal ICH cannot be based on ultrasonographic findings alone because it has low sensitivity for minor hemorrhages, and MRI scan will be helpful in establishing the diagnosis as a valuable complementary tool with more accuracy.^[2]^ In our case, the diagnosis of fetal ICH was primarily detected by ultrasound evaluation, and intra-uterine MRI aided in confirming the exact location of the hemorrhages, which were finally confirmed by autopsy.

Intracerebral hemorrhage accounts for over 80% of fetal ICH, carries very high morbidity and mortality rates, and occurs in less than 20% of patients.^[3]^ To date, only 1 case of combined intracerebral and subdural hemorrhage has been reported,^[3]^ and this case was added to the previous case and provided further evidence for multifocal hemorrhagic lesions.

The etiology of fetal ICH has not been firmly established, and predisposing factors such as infectious disease, maternal trauma, drug exposure, seizures, hypoxia, immune thrombocytopenia, coagulation disorders, twin-to-twin transfusion syndrome, demise of a co-twin in monochorionic placentation, fetal thrombophilia, cord entanglement, and fetal alloimmune thrombocytopenia have been attributed to the cause.^[2,7]^ When common causes of fetal ICH cannot be identified, genetic disorders^[8,9]^ such as COL4A1 and COL4A2 should be considered.^[8]^ From the available evidence, our patient had none of the known risk factors mentioned above, but the disease history hidden by the patient cannot be completely ruled out, and this is difficult to prove. A previous study suggested that a decreased PI of the middle cerebral artery may contribute to antenatal hemorrhage,^[4]^ but a mildly decreased PI in our case is unlikely to cause severe bleeding. Moreover, cerebrovascular malformations have been reported as extremely rare conditions that may lead to ICH; however, the diagnosis of malformations relies on contrast-enhanced CT or MRI, and intravascular contrast media are contraindicated in pregnant women.

According to the available data, the prognosis of fetal ICH generally depends on the grade of hemorrhagic lesions. ICH classified as grade III or grade IV appears to have a poorer prognosis than grade I or grade II.^[3,5,6]^ However, given the limited number of reported cases, it is difficult to establish firm prognostic figures. In our case, owing to the unfavorable prognosis, prompt termination of pregnancy was appropriate, although there is currently no consensus on the optimal mode of delivery. When fetal ICH is diagnosed, neonatology specialist counseling, neurological sequelae evaluation, and parental discussion are required.

## 5 Conclusion

Although the etiology of fetal ICH has been explored over the last decade, some cases remain medically unexplained. When massive and multifocal fetal ICH is confirmed, prompt termination of pregnancy is appropriate owing to the unfavorable prognosis.

## Acknowledgment

The authors wish to thank Dr. Fenglin Jia and Dr. Yanmei He for providing the original MRI images and reports.

## Author contributions

**Conceptualization**: Baorong Gao, Li Zhang.

**Drafting the manuscript:** Baorong Gao.

**Resources:** Qiang Wei.

**Writing – original draft:** Baorong Gao, Li Zhang.

**Writing – review & editing:** Li Zhang.

**Writing – review and language polish:** Li Zhang.
